# Fault Detection Approach of Cyclotron Ion Sources Based on KPCA-ISSA-SVM

**DOI:** 10.3390/s26082336

**Published:** 2026-04-10

**Authors:** Yunlong Li, Yuntao Liu, Fengping Guan, He Zhang, Shigang Hou, Peng Huang, Zhujie Nong

**Affiliations:** 1 Department of Nuclear Technology and Application, China Institute of Atomic Energy, Beijing 102413, China; 2 China lsotope & Radiation Corporation, Beijing 100089, China

**Keywords:** cyclotron, ion source, fault detection, Support Vector Machine, Sparrow Search Algorithm, Kernel Principal Component Analysis

## Abstract

To address the challenges of difficult feature extraction and suboptimal parameter configuration for cyclotron ion source fault diagnosis in complex environments, this study proposes an intelligent diagnostic framework integrating Kernel Principal Component Analysis (KPCA), an Improved Sparrow Search Algorithm (ISSA), and a Support Vector Machine (SVM). The KPCA algorithm is employed for dimensionality reduction to handle the highly nonlinear nature of fault data. Regarding algorithmic evolution, the basic SSA is enhanced by integrating dynamic weights, opposition-based learning, and Cauchy mutation strategies, which effectively overcome the diagnostic bottlenecks inherent in cyclotron scenarios. Furthermore, the ISSA facilitates the global adaptive optimization of key SVM parameters, eliminating the stochasticity of empirical tuning and fundamentally enhancing the model’s robustness. Experimental results across 30 independent tests demonstrate that the KPCA-ISSA-SVM model achieves an average accuracy of 97.6% in multi-class fault detection. Compared with other classic diagnostic models, the proposed framework exhibits superior precision and stability, providing an effective technical approach with significant engineering value for the precise monitoring of ion source statuses.

## 1. Introduction

The applications of nuclear technology expand from energy to various fields such as medicine [1], high-end manufacturing [2], and food safety [3], exhibiting a trend of rapid growth. In this context, the cyclotron serves as a representative example of nuclear technology with a wide range of applications. In nuclear medicine, Positron Emission Tomography (PET) is commonly used for the early diagnosis of tumors, neurological disorders, and cardiovascular diseases. Over 90% of the radionuclides, which are the core raw materials for PET, are produced by cyclotrons [4]. The growing demand for precision medicine imposes stricter requirements on short-lived isotopes, driving the development of compact, high-intensity, and serialized cyclotrons. In therapeutic applications, Boron Neutron Capture Therapy (BNCT) employs a cyclotron as a neutron source [5]. High-energy protons bombard a target to generate epithermal neutron beams [6], which trigger targeted nuclear reactions to eradicate tumor cells. Furthermore, in high-end manufacturing, these proton beams facilitate radiation hardening verification for aerospace components and single-event effect testing for semiconductors. These applications significantly shorten research and development cycles and improve device reliability.

Stable cyclotron operation is critical in the aforementioned scenarios. It directly influences radionuclide yield, the quality of clinical diagnosis and treatment, and the reliability of aerospace-grade components. Across all cyclotron applications, a stable beam remains essential [7]. As the core component of the cyclotron, the ion source system determines beam stability. During operation, ion source anomalies and severe power supply sparking account for a significant portion of beam downtime [8]. The causes of these abnormal states are categorized into internal and external factors. Internal factors include beam outages caused by mismatched parameter settings, filament aging, insufficient vacuum pressure, and inadequate gas purity. External factors primarily involve environmental interferences, such as ambient temperature, humidity, magnetic field fluctuations, and grounding integrity. A fault in the ion source leads to the interruption of the entire cyclotron operation [9]. If these problems are not diagnosed and addressed promptly, they can result in irreversible losses. Therefore, implementing effective monitoring and fault identification for the ion source system is of great significance for the stable operation of the accelerator.

Currently, the status of cyclotron ion source systems is primarily managed through a “post-fault detection and analysis” mode [10,11,12,13]. First, key components such as the ion source, arc chamber, and extraction electrode are inspected and analyzed after the cyclotron operation ends, according to a preset maintenance cycle. Second, fault localization and repair are performed only after macro-failures. These failures include a sudden drop in beam current, a plunge in arc voltage, or an extraction power anomaly. They also occur when interlock mechanisms trigger a shutdown.

Wei analyzed the installation position and vacuum chamber of the ion source [14]. The study found that reducing beam intensity and extending operation time while meeting beam requirements could decrease faults in the ion source and related systems. Calvo et al. built a compact cyclotron ion source test bench [15]. They systematically compared the effects of arc current, magnetic field, and gas pressure on the yield of negative hydrogen ions and derived the operating characteristics of the ion source, which provided guidance for cyclotron operation and maintenance. Mamedov et al. analyzed the ignition conditions of gas discharge on the outer cathode of a Penning ion source through numerical simulations and experimental research [16]. They summarized a scheme to prevent side discharge ignition, which effectively improved the lifespan of the ion source.

While the aforementioned strategies maintain the ion source to some extent, they lack comprehensive monitoring across the entire operational cycle. This cycle covers arc discharge startup, beam extraction, and stable beam delivery. Consequently, fault traceability is delayed. It becomes difficult to identify early performance degradation and potential failure mechanisms. Furthermore, fault handling mainly relies on manual experience and simple signal monitoring. It usually requires cooperation among personnel from different systems. They must troubleshoot the power supply, control, beam diagnostic, and vacuum systems one by one. This process consumes significant time and manpower, resulting in low efficiency. Furthermore, the operating environment of the cyclotron restricts parameter monitoring, making it impossible to track all system parameters. This limitation, along with a large amount of interference data, imposes higher requirements on fault detection technology [17,18,19,20,21].

With the rapid development of artificial intelligence, data-driven intelligent detection technology is now applied to ion source systems [22]. Building multi-parameter monitoring and intelligent detection models is essential to compensate for the time lag of traditional offline diagnosis. It also addresses the inherent problem of strong coupling between multiple cyclotron systems. This approach has become a key technology for improving ion source stability and cyclotron reliability.

Wang et al. proposed an ion beam stability prediction method based on a TCN-DTW network to address the issue of beam intensity fluctuations in ECR ion sources [23]. This method effectively predicts the beam trend of ECR ion sources and provides a technical solution for improving the long-term operational stability of heavy-ion accelerators, helping to reduce accelerator efficiency loss and prevent distortion of experimental terminal data. Kong et al. developed a real-time fault detection method based on SVM fusion [24]. This method addressed the challenges of fault identification in high-dimensional and nonlinear data from cyclotron ion sources and beamline systems. Their model achieved an accuracy exceeding 90% and effectively enhanced system reliability.

The ion source system is the foremost and core component for beam generation in a cyclotron. Its status directly affects beam quality [25]. The operating parameters of this system exhibit high dimensionality, strong coupling, and nonlinearity [26]. Traditional fault identification methods based on thresholds or offline spot checks struggle to meet real-time and accuracy requirements. Complex scenarios involve high-dimensional variables, intensive noise, and multiple coupled fault modes. Existing studies do not fully resolve critical issues such as feature redundancy and fuzzy classification boundaries. Consequently, there is an urgent need for more robust nonlinear feature extraction and intelligent classification strategies. This exploration will provide new methodological support for ion source fault diagnosis.

Based on the 14 MeV cyclotron at the China Institute of Atomic Energy (CIAE), this study proposes a fault detection method for the ion source system using SVM. Since the ion source system involves numerous parameters with nonlinear relationships [27], KPCA is utilized for feature extraction. This process reduces redundant information within the data. The processed low-dimensional data are then trained through an established SVM network to determine whether the input signals are normal. To improve recognition performance, the ISSA is employed to optimize SVM penalty parameters and kernel function parameters. This approach resolves the sensitivity issues associated with traditional SVM hyperparameters. Finally, the proposed model is applied to the 14 MeV cyclotron ion source system for verification. The results demonstrate that the model can efficiently and accurately identify common faults. It assists operators in quickly locating fault sources and significantly enhances the operational stability of the cyclotron.

The rest of this paper is organized as follows. Section 2 introduces the fault detection problem of the cyclotron ion source and details the fault detection method based on the SVM. The method includes KPCA, SVM principles, and the basic SSA. Section 3 proposes specific strategies for improving SSA to optimize SVM. Section 4 discusses the fault recognition results. It covers the ion source system composition, diagnostic schemes, operation status identification, and experimental verification of multi-type fault recognition. Finally, conclusions are drawn in Section 5.

## 2. Fault Detection Method

### 2.1. Fault Detection Problem of the Cyclotron

For cyclotron, the purpose of fault detection is to identify potential issues and provide early warnings to operators [28]. This process effectively protects equipment and ensures personnel safety. During operation, many factors affect cyclotron stability. It is difficult for humans to quickly judge and adjust to subtle changes. Characterizing cyclotron operation through physical modeling faces significant challenges due to space charge effects, coupling effects between different systems, and dynamic feedback from beam-loaded power supplies.

In this study, a data-driven method using cyclotron operation data is adopted. This method effectively addresses issues such as the curse of dimensionality, unknown boundary conditions, and the exponential amplification of initial errors. Instead of using differential equations for source tracing, the cyclotron operating state is treated as a “black box.” Key operating parameters are subsequently used to train the model. The trained model then maps these parameters to corresponding “normal” or “fault” labels to complete the fault detection of the cyclotron state. In this classification problem, the cyclotron input samples are defined as feature vector A, and the output samples are the given labels of class Y. A classification model is trained to map features to specific labels, which is represented by F(·). The feature vector of the input sample, $A={\left[a_{1},a_{2},a_{3},\ldots ,a_{n}\right]}^{T}$, is processed through F(·) to determine the category $Y=[Y_{1},Y_{2},Y_{3},\ldots ,Y_{m}]$. The classification detection of cyclotron input data is expressed as:

(1) $$F_{i}(A)\geq F_{j}(A),\;\;i\neq j,\;\;i,j=1,2,\cdots ,m$$

Each category $Y_{i}$ corresponds to a discriminant function $F_{i}(A)$. The feature vector A generates a response value through $F_{i}$, and the category of A is determined by the maximum response value. In the feature space, the cyclotron operating states are classified based on different input features. The boundaries between these classes represent the decision boundaries of the model, as shown below:

(2) Fi(A)−Fj(A)=0,  i,j=1,2,⋯,m

The decision boundary acts as a hypersurface that partitions class $Y_{i}$ and class $Y_{j}$ in the feature space. The normal vectors on both sides of different class boundaries provide a measure of the confidence level for the classification. Samples A closer to the boundary are more susceptible to being classified into incorrect regions by noise. The geometric position and curvature of the decision boundary directly affect the risk of misclassification and the robustness of the model. Thus, the decision boundary plays a critical role in mapping the cyclotron input feature vector A to the category Y.

### 2.2. Kernel Principal Component Analysis

In addition to its internal factors, the working status of the cyclotron ion source involves environmental conditions and other systems [29]. These variables couple with each other and exhibit complex nonlinear characteristics, making it difficult to extract key information. PCA performs a basis transformation in the original linear space and relies on a second-order covariance matrix to extract principal components [30]. It handles linearly separable high-dimensional data well. However, when the cyclotron beam status shows strong nonlinear coupling with physical quantities such as vacuum, gas flow, and thermal fields, linear projection inevitably causes the aliasing and loss of principal components. Therefore, KPCA is adopted to introduce various kernel functions tailored to the specific characteristics of the input data. By implicitly mapping the data into a high-dimensional space, this process unfolds originally inseparable low-dimensional data into a linearly separable structure. Then, PCA is applied in this high-dimensional space to reduce dimensionality and retain the main components [31,32].

Suppose the cyclotron ion source operation dataset is defined as A. It includes p-related variables, and each variable contains q samples. The ion source fault detection dataset is expressed as:

(3) $$A=[\begin{array}{cccc} \begin{array}{c} a_{11}\\ a_{21}\\ \vdots \\ a_{q1} \end{array} & \begin{array}{c} a_{12}\\ a_{22}\\ \vdots \\ a_{q2} \end{array} & \begin{array}{c} \cdots \\ \cdots \\ \cdots \end{array} & \begin{array}{c} a_{1p}\\ a_{2p}\\ \vdots \\ a_{qp} \end{array} \end{array}]=[\begin{array}{c} a_{1}\\ a_{2}\\ \vdots \\ a_{q} \end{array}]$$

where the sample $a_{i}$ is a p-dimensional vector belonging to the space $R^{p}$, representing the observed values of p-related variables in the i-th sampling, and q is the total number of samples.

The two-dimensional dataset is then transformed into a high-dimensional space H via a kernel function:

(4) $$\phi :R^{p}\to H,\;a_{i}\to \phi (a_{i})$$

The covariance matrix of the fault data mapped into the high-dimensional space H is expressed as:

(5) $$S^{H}=\frac{1}{q}\sum_{i=1}^{q}\phi (a_{i})\phi {\left(a_{i}\right)}^{T}=\frac{1}{q}\phi \phi^{T}$$

Since the mapping ϕ in Equation (5) cannot be explicitly defined, the value of $\phi (a_{i})\phi {\left(a_{i}\right)}^{T}$ cannot be calculated directly. Therefore, an eigenvalue decomposition is performed on the covariance matrix. Let λ be the eigenvalue and V be the eigenvector matrix. Its update equations are as follows:

(6) $$\lambda V=S^{H}V$$

Considering the case where the eigenvalue λ is non-zero, dividing both sides of Equation (6) by λ yields the projection of the mapped data $\phi (a_{i})$ in the direction of the eigenvector. After reorganization, the eigenvector V can be represented as a linear combination of the mapped sample vectors $\phi (a_{i})$:

(7) $$V=\sum_{i=1}^{q}\beta_{i}\phi (a_{i})$$

where $\beta_{i}$ is a *q*-dimensional vector, $\beta_{i}={\left[\beta_{1},\beta_{2},\ldots \beta_{q}\right]}^{T}$.

The matrix K is defined as the kernel matrix, which is calculated through the specified kernel function:

(8) $$K_{i,j}={\left[\phi {\left(a_{i}\right)}^{T}\cdot \phi (a_{j})\right]}_{q\times q}$$

The aforementioned derivation is conducted under the assumption of non-zero eigenvalues. To account for other cases, eliminate the influence of the data mean and correct offsets in the feature space, and centering the calculated kernel matrix is necessary. This ensures that principal component analysis accurately captures the covariance structure of the fault data [33,34]. The centering equation is expressed as follows:

(9) $$\bar{K}=K-I_{q}K-kI_{q}+I_{q}KI_{q}$$

where $I_{q}$ is a q×q matrix.

The x-th principal component of the cyclotron operation data samples is calculated as:

(10) $$t_{x}=\sum_{i=1}^{q}\nu_{i}^{x}\bar{K}(a_{i},a)$$

where $v_{i}^{x}$ denotes the i-th element of the x-th feature vector $V_{x}$.

An eigenvalue decomposition is performed on the centered kernel matrix:

(11) $$\bar{K}=VDV^{T}$$

where V is the corresponding eigenvector matrix, and $D=\mathrm{diag}(\lambda_{1},\lambda_{2},\ldots ,\lambda_{q})$ is a diagonal matrix and $\lambda_{1}\geq \lambda_{2}\geq \ldots \geq \lambda_{q}$. The diagonal elements represent the eigenvalues.

The contribution rate of the x-th principal component is:

(12) $$PV=\lambda_{i}/\sum_{j=1}^{q}\lambda_{j},\;i=1,2,\ldots$$

To retain the primary information of the original data and achieve dimensionality reduction, the eigenvalues are sorted in descending order. The first N eigenvalues are selected based on the cumulative contribution rate to satisfy the following condition:

(13) $$\sum_{i=1}^{N}PV_{i}\geq Per$$

where Per is the threshold for the cumulative contribution rate.

In fault detection, this threshold is typically selected between 85% and 95% to dynamically determine the number of retained principal components. Finally, the eigenvectors corresponding to the N largest eigenvalues constitute the reduced-dimension feature space, which is used for subsequent fault identification and classification.

### 2.3. Support Vector Machine

Due to the limited direct monitoring parameters for the ion source system in cyclotrons, the scarcity of fault samples and their complex distribution boundaries pose significant challenges to fault detection. Therefore, this study adopts the SVM as the core classifier. After processing with the KPCA algorithm, the original high-dimensional, noisy ion source operation data is mapped into principal component vectors within a low-dimensional feature space. The processed data retains the main variance contributions and eliminates correlations between variables, thereby providing high-quality input features for the SVM algorithm.

The core principle of SVM is to minimize structural risk, which involves compressing model complexity with limited samples before further reducing training errors [35,36]. Compared to neural networks, SVM effectively solves fault discrimination problems characterized by high dimensionality, small sample sizes, and non-convex distributions. It demonstrates superior generalization capability under limited data conditions and efficiently addresses high-dimensional recognition tasks involving small samples, nonlinearities, and interference.

Assume a fault sample set $T=\{(a_{i},y_{i})\},i=1,2,\ldots ,N$, where $a_{i}\in R^{N}$ is the input feature vector after KPCA feature extraction, and $y_{i}\in \{-1,+1\}$ is the corresponding fault category label. The primary objective of SVM classification is to identify an optimal hyperplane within the feature space. This hyperplane is designed to partition samples of different categories while simultaneously maximizing the margin between them. The partitioning equation can be expressed as:

(14) $$w^{T}\cdot \varphi (a)+b=0$$

where w is the normal vector of the hyperplane, b is the bias, and φ(a) represents the nonlinear mapping function that transforms data samples into the feature space.

To compensate for classification errors made by the model, slack variables $\xi_{i}$ and a penalty factor C are introduced to enhance the SVM’s classification performance. After incorporating these new parameters, the problem is formulated as the following optimization task:

(15) $$\min_{w,b,\xi }\frac{1}{2}\|w{\|}^{2}+C\sum_{i=1}^{N}\xi_{i}$$

(16) $$s.t.\;y_{i}(w^{T}a_{i}+b)\geq 1-\xi_{i},\;\xi_{i}\geq 0,\;i=1,\ldots ,N$$

In the objective function, min denotes the minimization of the target function to find the optimal value. The term $1/2(\|w{\|}^{2})$ is the regularization term, and C is the penalty factor that controls the model’s tolerance for misclassification. $\sum_{i=1}^{N}\xi_{i}$ represents the sum of slack variables, where $\xi_{i}$ indicates the degree of misclassification for the i-th feature vector.

Equation (16) serves as the constraint condition to ensure the correct classification of each feature vector $a_{i}$. When $\xi_{i}=0$, the feature vector lies strictly on one side of the decision boundary; when $\xi_{i}>0$, the feature vector is located within the slack region, allowing for a certain degree of misclassification.

To efficiently solve the aforementioned constrained optimization problem, Lagrange multipliers $\alpha_{i}={\left[\alpha_{1},\alpha_{2},\ldots ,\alpha_{N}\right]}^{T}$ are introduced to construct the Lagrange function, transforming the primal problem into its dual problem:

(17) $$\max_{\alpha }\sum_{i=1}^{N}\alpha_{i}-\frac{1}{2}\sum_{i=1}^{N}\sum_{j=1}^{N}\alpha_{i}\alpha_{j}y_{i}y_{j}K(a_{i},a_{j})$$

(18) $$s.t.\sum_{i=1}^{N}\alpha_{i}y_{i}=0,\;0\leq \alpha_{i}\leq C,\;i=1,\ldots ,N$$

where $K(a_{i},a_{j})$ is the kernel function.

To address the nonlinear distribution characteristics of cyclotron data, the Radial Basis Function (RBF) kernel is employed, and its expression is as follows:

(19) $$K(a_{i},a_{j})=\exp(-\frac{\|a_{i}-a_{j}{\|}^{2}}{2\sigma^{2}})$$

where σ is the kernel parameter that controls the complexity of the model boundary, $g=1/2\sigma^{2}$.

After solving the aforementioned quadratic programming problem to obtain the optimal solution $\alpha^{*}$, the final fault classification decision function is given by:

(20) $$f(x)=\mathrm{sign}\;(\sum_{i=1}^{N}\alpha_{i}^{*}y_{i}\exp(-\frac{\|a_{i}-x{\|}^{2}}{2\sigma^{2}})+b^{*})$$

where x is the test sample, $a_{i}$ is the training sample, and the physical significance of the decision function is the Euclidean distance between the two.

Since cyclotron ion source fault detection is a multi-classification problem with various fault types, while the standard SVM model is typically a binary classifier, the strategy is extended to one-vs-one classification. For k types of faults, k(k−1)/2 classifiers are constructed to perform pairwise decision-making and determine the final fault category.

### 2.4. Sparrow Search Algorithm

In traditional SVM applications, the selection of hyperparameters is crucial to model performance. However, this selection often relies on empirical judgment, which may lead to poor classification results or difficulty in finding the optimal solution [37]. To address this, the Sparrow Search Algorithm (SSA), which is a meta-heuristic algorithm, is introduced for optimization. Proposed by Xue et al. in 2020, SSA is a novel intelligent optimization algorithm that simulates the foraging and anti-predatory behaviors of sparrows in nature [38]. It features an intelligent search for optimal solution sets and strong stability.

In the SSA, each sparrow is an agent, and the agents are divided into three categories: discoverers, followers, and defenders. Assuming the number of agents is N and the dimension of the solution space is d, this paper mainly uses it to optimize the SVM hyperparameters C and g, thus d=2. The position vector of the i-th agent at the t-th iteration is represented as $X_{i}^{t}=[x_{i,1}^{t},x_{i,2}^{t},\ldots ,x_{i,d}^{t}]$, and its corresponding fitness value $f(X_{i})$ represents the SVM classification accuracy.

Discoverers are primarily responsible for the optimization direction of the population, accounting for a small portion of the total agents, generally less than 30%. The position update is described as:

(21) $$x_{i,j}^{t+1}=\left\{\begin{array}{ll} x_{i,j}^{t}\cdot \exp(-\frac{i}{\alpha \cdot T_{max}}), & \mathrm{if}\;R_{2}<ST\\ x_{i,j}^{t}+Q\cdot L, & \mathrm{if}\;R_{2}\geq ST \end{array}\right.\;$$

where t is the current iteration number of the algorithm, and $T_{max}$ is the maximum number of iterations, α∈(0,1] is a random number used to control the search range of the discoverers, Q is a random number following a normal distribution, L is a 1×d matrix, $R_{2}$ and ST simulate the environmental state of sparrows in the wild, $R_{2}\in [0,1]$, and ST∈[0.5,1.0].

If a discoverer finds a better fitness value in the solution space, the followers will search around the discoverer; otherwise, the discoverer will update randomly in the solution space, and its position update equation is:

(22) $$x_{i,j}^{t+1}=\left\{\begin{array}{ll} Q\cdot \exp(\frac{x_{worst}^{t}-x_{i,j}^{t}}{i^{2}}), & \mathrm{if}\;i>N/2\\ x_{P}^{t+1}+|x_{i,j}^{t}-x_{P}^{t+1}|\cdot A^{+}\cdot L, & \mathrm{if}\;i\leq N/2 \end{array}\right.\;$$

where $x_{P}$ denotes the optimal position currently held by a discoverer, $x_{worst}$ represents the current global worst position, A is a 1×d matrix with elements randomly set to 1 or −1, and $A^{+}=A^{T}{\left(AA^{T}\right)}^{-1}$.

When i>N/2, it indicates the follower is in a low-fitness position and must fly to other areas according to the equation. Conversely, the follower performs a local search in the vicinity of the discoverer.

Defenders simulate the behavior of sparrows evading predators in the environment to enable the algorithm to escape local optima. Generally accounting for approximately 15% of the total agents, they are updated according to the following equation:

(23) $$x_{i,j}^{t+1}=\left\{\begin{array}{ll} x_{best}^{t}+\beta \cdot |x_{i,j}^{t}-x_{best}^{t}|, & \mathrm{if}\;f_{i}>f_{g}\\ x_{i,j}^{t}+K\cdot (\frac{\left|x_{i,j}^{t}-x_{worst}^{t}\right|}{(f_{i}-f_{w})+\varepsilon }), & \mathrm{if}\;f_{i}=f_{g} \end{array}\right.\;$$

where $x_{best}$ represents the current global optimal position of the agents, β follows a standard normal distribution and is used to control the search step size of the agents in the solution space, K∈[−1,1] is a random number that controls the movement direction of the agents in the solution space, and $f_{i}$, $f_{g}$, and $f_{w}$ represent the agent’s current, globally optimal, and globally suboptimal fitness values, respectively.

## 3. ISSA Optimization for SVM

### 3.1. Improvement of Agent Population Initialization

In the standard SSA, agent initialization is randomly distributed within the solution space, generating N initial position vectors $X_{i}$ of d dimensions. However, this random distribution involves uncertainty, which often leads to an uneven distribution of agents. Consequently, the optimization performance and efficiency of the algorithm are compromised in the later stages. To address this issue, a chaotic map is introduced to refine the population initialization. This approach improves the uniformity of the agent distribution within the solution space, as formulated below:

(24) $$x_{i+1}=\left\{\begin{array}{ll} \cos(\pi \cdot \left[2rx_{i}+4(1-r)x_{i}(0.5-x_{i})\right]), & x_{i}<0.5\\ \cos(\pi \cdot \left[2r(1-x_{i})+4(1-r)x_{i}(0.5-x_{i})\right]), & x_{i}\geq 0.5 \end{array}\right.$$

where r∈[0,1] represents the control parameter, and cos denotes the cosine function utilized to map the results into the range of [0,1].

In Equation (24), a composite mapping method is adopted, integrating piecewise characteristics, nonlinear mapping, and periodic folding. The initial distribution of agents in the original Sparrow Search Algorithm (SSA) is entirely random, which may lead to uncertainty in search efficiency. By controlling the value of r, the improved chaotic mapping regulates the degree of randomness in agent distribution. This ensures that agents are uniformly distributed throughout the solution space during initialization.

**Figure 1:**
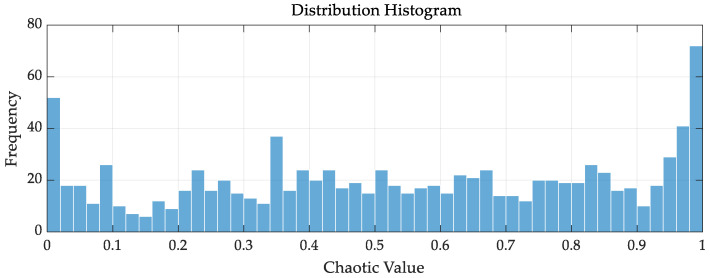
Initial distribution histogram of the improved chaotic mapping.

Figure 1 illustrates the initial distribution of the improved chaotic mapping, which simulates the random distribution of 1000 agents within the range [0, 1]. Following extensive experiments, the value of r is determined to be 0.72, at which point the chaotic sequence exhibits excellent ergodicity and randomness.

During the initial stage, the algorithm effectively captures the boundary regions of the solution space. This capability compensates for the tendency of the standard SSA to fall into local optima when handling complex boundary constraint problems, increasing the probability of finding the global optimum. Although the boundary weights are relatively large, the histogram distribution in the middle region remains stable and continuous. This equilibrium ensures that no significant blind spots exist for the agents within the entire search interval, allowing the algorithm to establish a highly diverse initial population during early iterations.

### 3.2. Dynamic Adaptive Weight Strategy

In the discovery phase of the SSA, the position update of agents relies on exponential decay, which poses a risk of instability. To refine the discovery phase described in Equation (21), a nonlinearly decreasing weight coefficient ω that varies with the iteration count is introduced. The improved equation is expressed as follows:

(25) $$\omega (t)=\tanh(2\cdot (1-\frac{t}{T_{max}}))$$

Based on the improvement of the ω weight coefficient, when $R_{2}<ST$, the position update equation for the agents is updated according to the current best fitness position and the current position via the weight coefficient, as formulated below:

(26) $$x_{i,j}^{t+1}=x_{i,j}^{t}+\omega (t)\cdot (x_{best}^{t}-x_{i,j}^{t})\cdot rand$$

**Figure 2:**
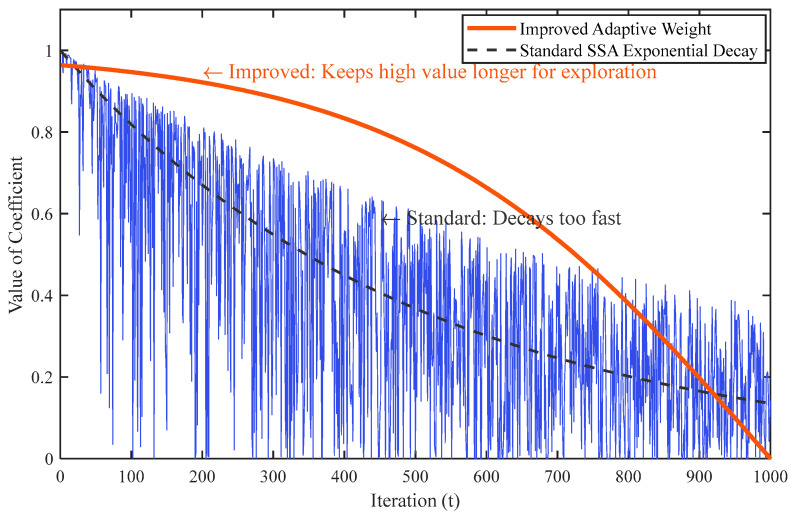
Comparison of coefficient curves between ISSA and SSA.

The comparison of coefficient curves between ISSA and SSA in the discovery phase is illustrated in Figure 2. In the SSA, the position decay curve of the agents decreases rapidly during the discovery phase, resulting in a lack of global optimization capability in the early stage of the algorithm. In the improved position update, the agents are guided by the current best position, which better balances global and local optimization capabilities. The coefficient ω is larger in the initial stage, allowing for exploration within a wider range of the solution space. As the algorithm progresses to the later stages and the decay speed increases, the agents can rapidly converge to the vicinity of the optimal solution.

### 3.3. Fusion of Opposition-Based Learning and Cauchy Mutation

To enhance the performance of followers, an improvement is made to Equation (22) by introducing opposition-based learning and Cauchy mutation. A new selection strategy is added, where the value of the selection factor $P_{s}$ determines which mutation strategy to employ. The selection probability is dynamically adjusted with the iteration count, as formulated below:

(27) $$P_{s}=-{\left(\exp(1-\frac{t}{T_{max}})\right)}^{20}+0.05$$

If $rand<P_{s}$, the opposition-based learning strategy is executed, performing the opposition operation on the best fitness position in the current solution space as follows:

(28) $$\begin{array}{l} {\tilde{x}}_{best}=ub+rand\cdot (lb-x_{best}^{t})\\ x_{new}={\tilde{x}}_{best}+b\cdot (x_{best}^{t}-{\tilde{x}}_{best}) \end{array}$$

where $b={\left(1-t/T_{max}\right)}^{t}$ is the convergence factor. An opposition-based solution is generated within the boundaries of the solution space, and b is utilized to control the degree of fusion between the current solution and the opposition-based solution. The search range is expanded to enhance the algorithm’s efficiency.

If $rand>P_{s}$, the Cauchy mutation strategy is executed according to the following equation:

(29) $$x_{new}=x_{best}^{t}+x_{best}^{t}\cdot \tan((rand-0.5)\cdot \pi )$$

where tan((rand−0.5)·π) represents the random number generated from the Cauchy distribution, which can introduce small perturbations near the global optimal value to assist the algorithm in local optimization. Meanwhile, the characteristics of the Cauchy distribution also enable a higher probability of mutation, helping the algorithm jump out of local optima.

### 3.4. SSA-SVM Fault Detection Process

The values of hyperparameters significantly influence the classification performance of the SVM. In this study, ISSA is employed to optimize these hyperparameters. By searching for an optimal combination of parameters (C,g), the ISSA-SVM model achieves the minimum fault diagnosis error rate on unknown datasets. To validate the model performance and effectively prevent overfitting, k-fold cross-validation is adopted for evaluation. The fitness function of ISSA is equivalent to the objective function of minimizing the classification error rate, as formulated below:

(30) $$\min f(C,g)=\frac{1}{k}\sum_{i=1}^{k}e_{i}(p)$$

where k is the number of folds for cross-validation, and $e_{i}(p)$ represents the error rate of the i-th fold in the cross-validation process.

**Figure 3:**
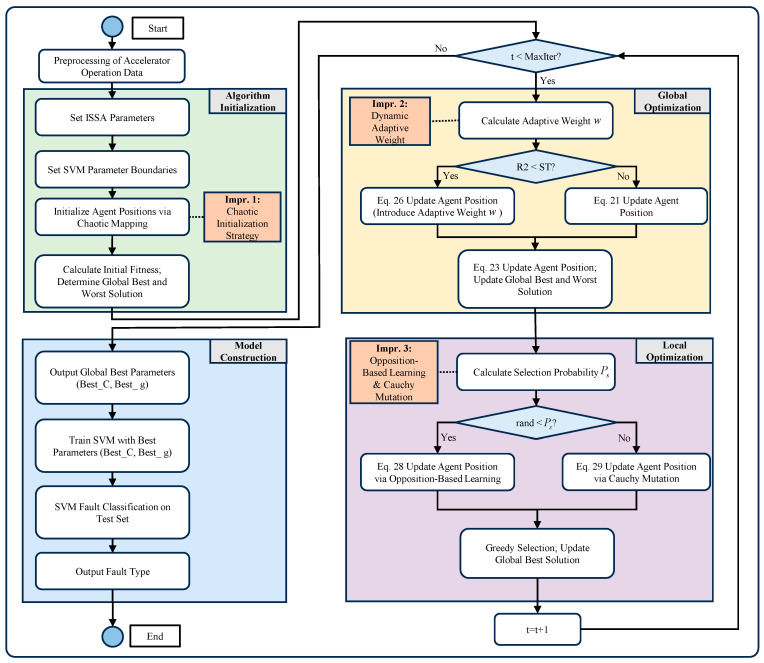
ISSA-SVM fault detection flow.

The SVM possesses excellent classification capabilities. In cases of limited samples, the key parameters C (penalty factor) and g (kernel parameter) are typically selected through empirical methods, which introduces significant uncertainty to the classification performance. ISSA is employed to optimize the SVM by adaptively selecting the optimal parameters to maximize classification effectiveness. The process is illustrated in Figure 3 and consists of four main modules:

(1) Algorithm Initialization: This module sets the model parameters and initializes the agents using the chaotic map described in Equation (24). This ensures a more uniform distribution in the initial space and enhances the global search capability of the model.
(2) Global Optimization Module: This module primarily focuses on global search. As shown in Equation (26), a dynamic adaptive weight ω is introduced to better balance the search proportions throughout the entire life cycle of the model.
(3) Local Optimization Module: Local search is refined through two entirely new strategies defined in Equations (28) and (29).
(4) Module Construction: The final module outputs the optimal combination of SVM parameters, which are then used by the SVM to classify fault types and output the detection results.

## 4. Results and Discussion of Fault Diagnosis

### 4.1. Ion Source System Composition

**Figure 4:**
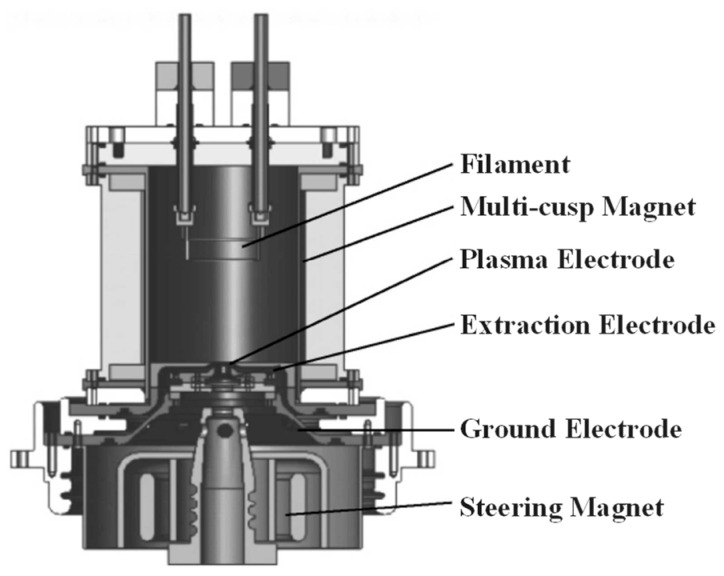
Structure of $H^{-}$ ion source.

The CIAE has established a serialized system of high-intensity cyclotrons, including 10 MeV, 14 MeV, 18 MeV, 30 MeV, 100 MeV, and 230 MeV models [39,40,41,42], all of which utilize $H^{-}$ ion sources. The structure of the ion source is illustrated in Figure 4.

The primary causes of high-voltage sparking in the ion source are categorized into structural constraints, vacuum environments, and operational factors. To meet the focusing requirements of beam optics, the design of the extraction and puller electrode region is compact with extremely small insulation gaps, leading to a significant increase in the electric field gradient in this area. During accelerator operation, the continuous injection of hydrogen gas reduces the local vacuum level at the extraction gap. Under such adverse conditions, the dielectric strength between the electrodes is greatly diminished, thereby triggering high-voltage breakdown phenomena.

**Figure 5:**
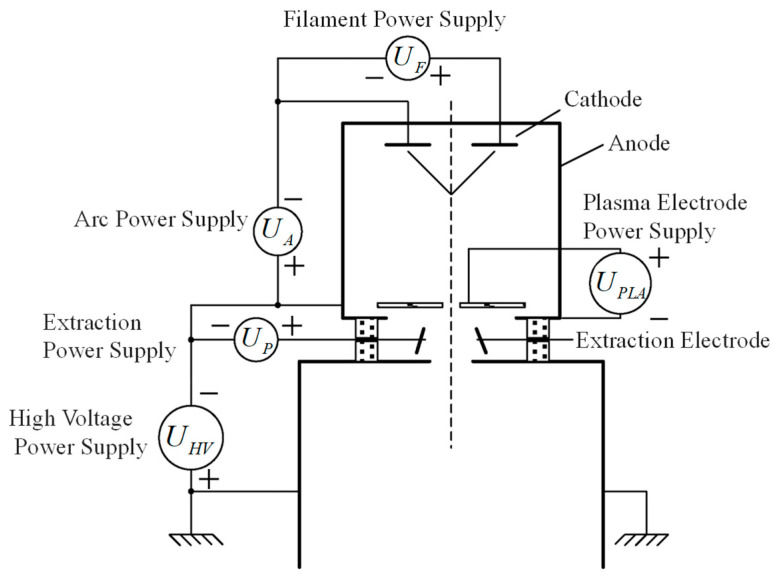
Relationship of ion source power supply potential.

The power supply system of the ion source consists of five sets of power supplies: filament power supply $\;U_{F}$, arc power supply $U_{A}$, plasma electrode power supply $U_{PLA}$, extraction power supply $U_{P}$, and high-voltage power supply $U_{HV}$. The power system adopts a high-voltage floating structure, using the ground potential as a reference, where $U_{HV}$ determines the reference negative high voltage. This design ensures independently adjustable potential differences between the electrodes, guaranteeing that the extracted ion beam is precisely tunable. This serves as the electrical foundation for the stable production of high-quality $\;H^{-}$. The potential relationships of the ion source power supplies are shown in Figure 5.

The measurement setup utilizes a Faraday cup to obtain the beam intensity and an emittance meter to detect the beam divergence. By controlling the matching relationships between the power supplies, a stable ion source beam current is output. A −40 kV high-voltage power supply is selected, while all other power supplies operate at high voltage potentials.

### 4.2. Technical Solution for Ion Source Fault Data Detection

**Figure 6:**
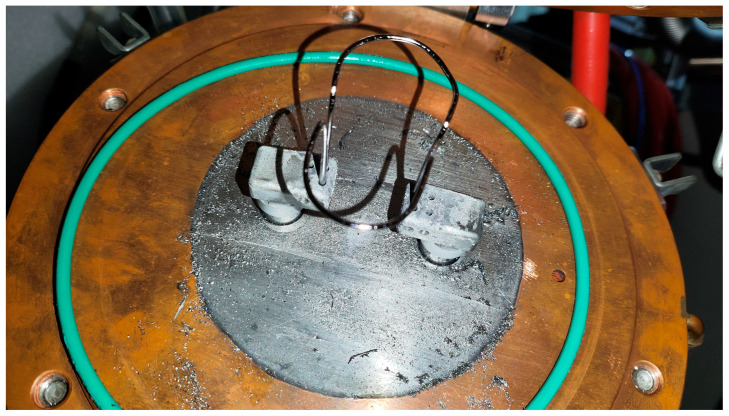
Deformation of the ion source filament and sputtered morphology on the upper cover plate following prolonged operation.

During actual long-cycle operation, the physical state of the core components within the ion source undergoes significant degradation over time. This degradation affects the matching characteristics between the power supplies and the load, as well as the stability of the beam. Figure 6 illustrates the state of the filament assembly and the top cover plate after the ion source completes an operating cycle. Under the combined influence of high temperature thermionic emission and the multipeak field, the large current flowing through the filament subjects it to continuous Lorentz forces and thermal stress. This may result in the twisting and deformation of the tungsten filament. This deformation not only alters the geometric center of electron emission and affects the spatial distribution of the plasma, but also potentially leads to changes in resistance as the filament thins, which causes a drift in the load characteristics of the filament power supply.

The surface of the ion source top cover is coated with a distinctive layer of metallic sputtering, which appears as a grayish-black powder. During ion source operation, a portion of the ions moving in the reverse direction bombards the electrodes and filament materials, causing the material to evaporate and deposit onto surrounding components, which leads to a significant decrease in dielectric strength. Under long-term high-voltage operating conditions, accompanied by severe sputter deposition, frequent high-voltage sparking occurs. In severe cases, this leads to power supply overcurrent protection or even a complete system shutdown.

Sparking faults are unavoidable during accelerator operation. If not addressed promptly, they not only cause equipment damage but also potentially lead to further safety risks and beam interruption. Relying solely on static power parameter matching cannot fully guarantee the long-term stability of the beam. Therefore, it is particularly necessary to establish a data-driven intelligent fault detection and prediction system targeting the aforementioned nonlinear fault characteristics (such as sparking and beam jitter) caused by physical wear.

**Figure 7:**
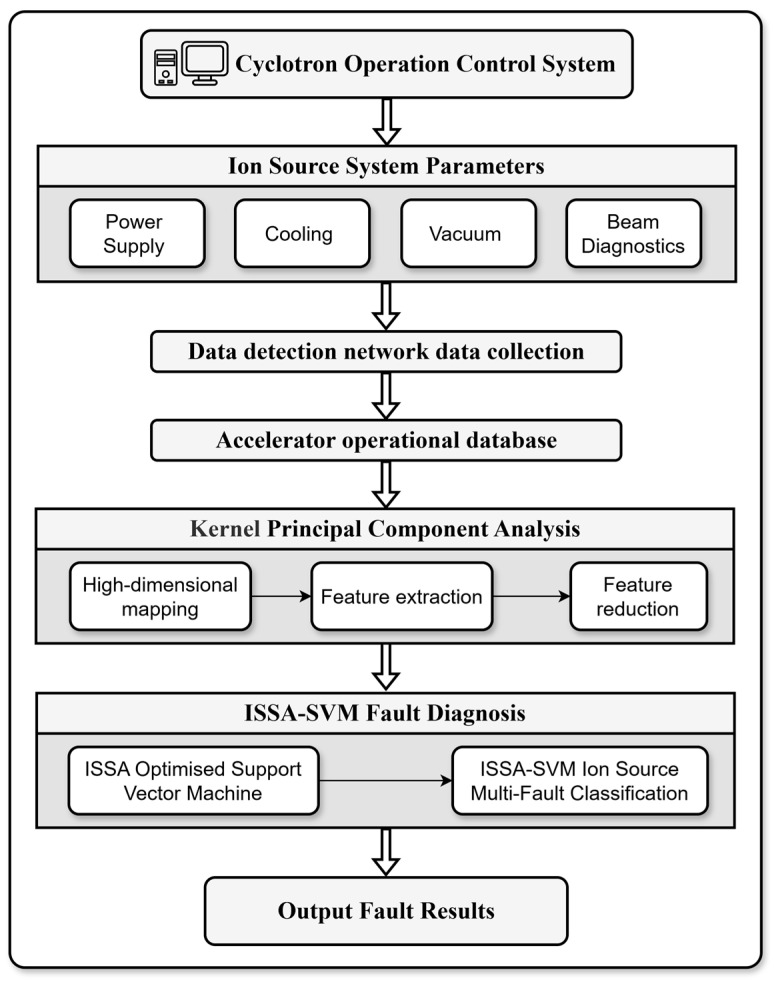
Technical scheme for ion source system fault detection.

The collected detection data from each system is processed using KPCA. Subsequently, an ISSA-SVM model is employed, utilizing ISSA to perform adaptive optimization of the key parameters for the SVM. The optimized model is then used to execute multi-fault classification for the ion source system, achieving precise identification of different fault modes. Finally, the diagnostic results are output. The technical scheme for the ion source system fault detection is illustrated in Figure 7.

The input features selected for the diagnostic model primarily include the voltage and current parameters of the power system, environmental temperature and humidity, gas flow rate, and vacuum level. Detailed parameters are listed in Table 1.

### 4.3. Ion Source Operating Status Detection

The 14 MeV cyclotron at the CIAE maintains a world-leading level of technology, achieving the extraction of a 1 mA high-intensity proton beam. During the experimental process, sparking faults occur in the ion source, which are unavoidable. Status identification is performed on the ion source data using SVM to distinguish whether the ion source is in a normal operating state or an abnormal fault state.

A basic binary classification SVM algorithm is employed. Several sets of typical values for C and g are selected to diagnose the accelerator status. The label “1” indicates normal operation, while “2” indicates a fault state. A total of 600 datasets are used, consisting of 300 normal operation datasets and 300 fault datasets. The training set accounts for 70%, and the test set accounts for 30%. There are 90 sets each for normal and fault data in the test phase. The fault identification results are shown in Table 2.

The dataset comprises samples collected from December 2023 to June 2025. The first 70% of the sequential data serves as the training set, while the remaining 30% constitutes the test set.

By comparing the results of the three groups, it is evident that the performance of the SVM model is highly dependent on the selection of parameters C and g. When smaller values are selected, the model exhibits weak generalization capability and fails to effectively distinguish between the normal and fault states of the ion source. Conversely, when larger values are selected, the classification boundary of the model becomes increasingly complex.

To verify the stability of the ISSA under different population sizes and ensure a fair comparison with the original SSA, four population scales (10, 30, 50, and 100) are set, with the maximum iterations fixed at 30. The training data remain consistent with the previous sections. The search range for the SVM penalty parameter C is [0.01,100], and the kernel function parameter g is $\left[2\times 10^{-5},\;2\times 10^{5}\right]$. The classification error rate is calculated according to Equation (30). Each configuration is executed independently five times to record the average optimization time (seconds), as well as the average, optimal, and worst-case values of the classification error rate. The results are summarized in Table 3.

As indicated in Table 3, the average classification error rate of the ISSA is consistently lower than that of the SSA across all population sizes. The optimal error rate of the ISSA reaches a minimum of $4.77\times 10^{-3}$, whereas the best result for the SSA is only $9.52\times 10^{-3}$. Although the execution time of both algorithms grows linearly with the increase in population size, the computational overhead of the ISSA remains slightly lower than that of the SSA. Notably, even with larger population sizes, the performance improvement of the SSA is limited, further confirming the inherent defect of the original SSA in falling into local optima. In contrast, the ISSA maintains stable and superior performance across different population scales, thanks to the integration of chaotic map initialization, dynamic weights, and other improvement strategies.

Table 4 evaluates the model performance under different numbers of iterations. With consistent parameters and datasets, the execution time for both algorithms shows an upward trend as the number of iterations increases. However, the computational overhead of the ISSA-SVM is consistently lower than that of the SSA-SVM, demonstrating higher efficiency. Regarding the classification error rate, the average, optimal, and worst-case values for the ISSA-SVM are significantly superior to those of the SSA-SVM across all iteration counts. When the number of iterations reaches 100, the performance of the ISSA-SVM remains stable, whereas the error rate of the SSA-SVM remains at a high level. These results further validate the effectiveness of the proposed improvement strategies.

**Figure 8:**
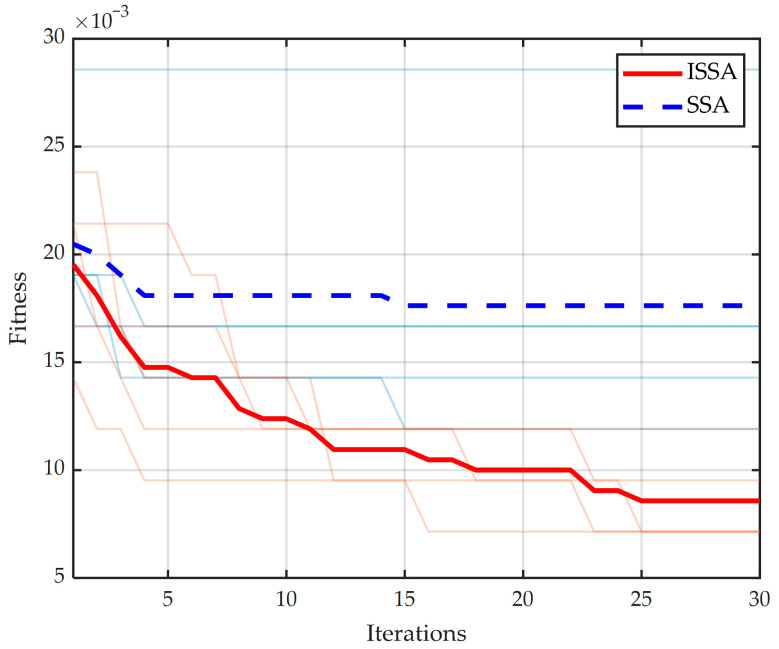
Comparison of fitness curves between SSA and ISSA for fault classification.

Figure 8 illustrates the optimization convergence curves of the algorithms before and after improvement. This optimization process aims to minimize the classification error rate, with both curves showing a clear downward trend. In the figure, the thin lines represent the original paths of five independent experiments, while the bold lines represent the average convergence levels. The data indicate that the SSA (blue dashed line) tends to fall into local optima during the early stages of iteration, with the final fitness value stabilizing around $17.6\times 10^{-3}$. In contrast, the ISSA (red solid line) exhibits a stronger capability to escape local extrema, and its fitness value continues to decrease, eventually converging to approximately $8.5\times 10^{-3}$. Furthermore, the five experimental curves of the ISSA are more tightly distributed, indicating higher algorithmic stability. These results fully demonstrate that the ISSA possesses superior global search capability and robustness.

According to the analysis of the fitness curves mentioned above, ISSA outperforms SSA in both parameter optimization accuracy and the ability to escape local extrema. To further verify the effectiveness of this optimization advantage in practical fault diagnosis, the optimal parameter combinations (C,g) corresponding to the end of the iterations for SSA and ISSA are extracted, respectively, and integrated into the SVM model for classification and prediction on the test set. The diagnostic results and specific performance indicators of SSA-SVM and ISSA-SVM on the actual test set are presented below.

**Figure 9:**
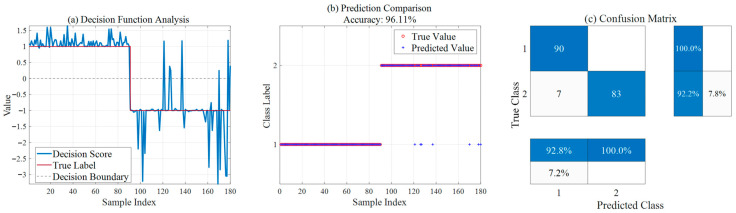
Ion source diagnosis results based on SSA-SVM.

**Figure 10:**
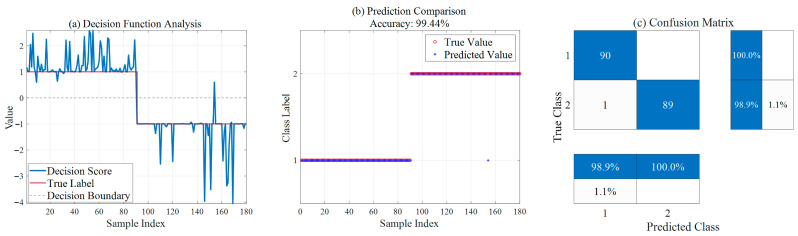
Ion source diagnosis results based on ISSA-SVM.

Table 5 presents the diagnostic results of the two models for 90 normal samples and 90 fault samples, respectively, intuitively illustrating the performance of SSA-SVM and ISSA-SVM in ion source status diagnosis. As shown in Figure 9, the SSA-SVM model achieves an overall accuracy of 96.11%. However, it exhibits deficiencies in identifying fault samples, with seven samples missing detection, resulting in a fault recognition rate of 92.2%. In contrast, the improved ISSA-SVM model, as shown in Figure 10, demonstrates excellent classification capability, increasing the overall accuracy to 99.44%. More importantly, the improved model significantly reduces the rate of missed fault detections; among the 90 fault samples, only one sample was misidentified, yielding a fault recognition rate as high as 98.9%. This validates the significant advantage of the ISSA in enhancing model generalization capability and diagnostic reliability.

### 4.4. Detection of Multi-Type Faults in Ion Sources

#### 4.4.1. Introduction to Ion Source Fault Types

To achieve refined monitoring and diagnosis of ion source operating status, the collected ion source sample data are categorized into six major classes based on the primary fault mechanisms and operating characteristics of the system. These categories cover the basic spectrum of the system, ranging from normal operation to various typical faults. Specifically, these include: normal operating samples, plasma sparking, extraction sparking, simultaneous plasma and extraction sparking, filament collapse sparking, and abnormal ion source status. The set of symbols {F1,F2,F3,F4,F5,F6} is adopted to encode these six states. The specific correspondences are presented in Table 6.

Fault types from F2 to F6 in the ion source are characterized by sudden onset and destructiveness. Once a fault occurs, it directly leads to the interruption of the accelerator beam, which severely impacts the experimental process. While plasma and extraction sparking can be recovered relatively quickly, filament power supply breakdown sparking triggers interlock sparking in other power supplies. This results in a longer recovery time and a significant impact on accelerator operation. Abnormal ion source status refers to anomalous surges in the load parameters of the control power supply. Although this does not lead to beam interruption, it degrades the beam quality and may further induce sparking issues in the ion source over a long period.

The selected data span a period of one year, during which multiple experiments are conducted. The ion source beam current ranges from 0.01 mA to 7 mA, and fault data from multiple experiments are selected for validation. For normal operating samples and abnormal ion source samples, parameters from the current operating period of the accelerator are selected. For sparking fault samples, data samples from the period immediately preceding the sparking event are chosen. It is difficult for human operators to determine whether these samples will lead to a fault. And it is also challenging to explain the operating conditions of such accelerators based solely on physical mechanisms, as these conditions involve multi-system parameters and change with the environment [43]. Therefore, the adoption of data-driven intelligent diagnostic methods becomes an effective way to solve this problem.

#### 4.4.2. Parameter Settings and Dimensionality Reduction

**Figure 11:**
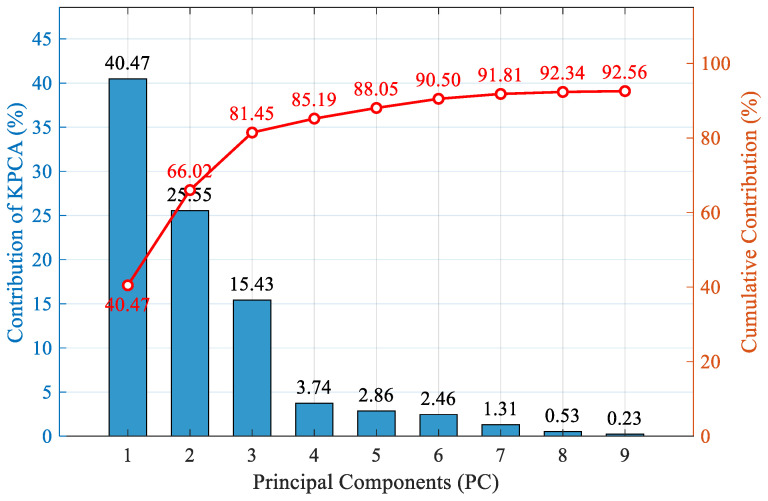
Cumulative contributions of principal components by KPCA.

To verify the performance of KPCA-ISSA-SVM in ion source fault identification, six categories of ion source data samples are selected, with 300 datasets for each category, totaling 1800 records. The ratio of training samples to validation samples is 7:3, meaning that 90 samples from each category are utilized for identification, resulting in a total of 540 samples for detection. The maximum number of iterations for ISSA is set to 30, and the population size is set to 10. The optimization range for the SVM parameter C is [0.1, 10^3^], and the kernel function parameter g range is [2 × 10^−5^, 2 × 10^5^]. The input data undergo dimensionality reduction via KPCA with a Radial Basis Function kernel, as defined in Equation (19). The reduced feature set is then fed into the ISSA-SVM diagnostic model, the architecture of which is illustrated in Figure 11.

The dataset comprises samples collected from December 2023 to June 2025. During this period, the 14 MeV cyclotron underwent various experiments, including chip testing, neutron imaging, and beam commissioning, during which the ion source remained in different operating statuses. The ion source beam current is approximately 10 μA during beam commissioning, whereas it varies from 0.01 mA to 7 mA in other experimental processes. Additionally, the ion source filament has a finite service life and was replaced multiple times during this period, with its wear level varying according to beam supply time and energy magnitude.

To avoid data leakage from future information, this study strictly follows the chronological order of time-series data for partitioning. In the initial stage, the total dataset is split into a training set (the first 70%) and a test set (the remaining 30%) based on the time sequence. Within the training set, a five-fold rolling-window validation is employed to construct the validation sets. Unlike traditional K-fold cross-validation, this method avoids random shuffling. Instead, it ensures that the validation set in each fold always follows its corresponding training set chronologically, thereby preserving the temporal dependency structure of the data.

The original input consists of 14 dimensions, which KPCA reduces to nine dimensions, achieving a total contribution rate of 92.56%. As the number of principal components increases, the cumulative contribution rate exhibits a trend of rapid initial ascent followed by a gradual leveling off. Among these, the first principal component accounts for the largest proportion of information at 40.47%, and the first three principal components contain over 80% of the information volume from the original high-dimensional feature space. When the first nine principal components are extracted, the cumulative contribution rate exceeds 90%. At this stage, the extracted principal components maximize the retention of key feature distributions from the original ion source fault data while effectively filtering out redundant features and environmental noise. Finally, the features after dimensionality reduction serve as inputs for the subsequent ISSA-SVM diagnostic model, reducing the computational complexity of the classification algorithm while ensuring feature integrity.

Table 7 summarizes the statistical results of five different dimensionality reduction algorithms over 30 independent runs. In this setup, the dimensionality-reduced data are processed by the ISSA-SVM model to evaluate the stability and effectiveness of each algorithm in the feature extraction process. The experimental results indicate that the KPCA algorithm significantly outperforms the other competing methods in overall performance. Specifically, KPCA achieves the highest average accuracy of 96.60% and an optimal accuracy of 99.63%. More importantly, the lowest accuracy of KPCA remains robust at 90.56%, whereas linear dimensionality reduction algorithms (such as LDA and PCA) and nonlinear methods (such as t-SNE) suffer from a drastic performance decline in their worst-case scenarios, with accuracies dropping below 52%. This contrast demonstrates the superior robustness and stability of KPCA in handling the nonlinear monitoring data of the ion source, effectively capturing critical features while suppressing noise interference. Regarding computational efficiency, the average execution time of KPCA is 6.56 s, which is slightly higher than that of PCA (6.22 s). However, considering the substantial gains in accuracy and stability, this computational overhead remains well within the acceptable limits for real-time diagnostic systems. In conclusion, KPCA represents the ideal dimensionality reduction technique for this ion source status recognition task.

#### 4.4.3. Multi-Fault Ion Source Diagnosis via KPCA-ISSA-SVM

The diagnosis utilizes the KPCA-ISSA-SVM model to process 90 datasets for each of the six ion source operating statuses defined previously. This process involves a total of 540 datasets encompassing categories F1 through F6. The model runs independently 30 times, and the optimal performance is recorded.

**Figure 12:**
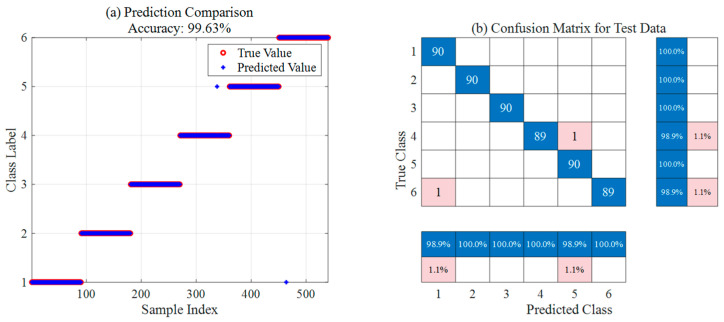
Optimal ion source diagnosis results based on KPCA-ISSA-SVM.

The diagnosis of the six defined ion source operating statuses via KPCA-ISSA-SVM achieves a classification accuracy of 99.63% on the test set. As observed intuitively from the prediction comparison plot in Figure 12a, the predicted values for the vast majority of test samples align perfectly with the true values, with only two exceptions. According to the confusion matrix analysis in Figure 12b, the model achieves a 100% recognition rate for categories 1, 2, 3, and 5. A misdiagnosis rate of 1.1% occurs within the category 4 and category 6 samples, where one sample from each is misidentified. Based on the normalized percentages across rows and columns for each category, the model demonstrates exceptionally high classification accuracy and robustness, enabling highly reliable intelligent diagnosis for multiple operating statuses of the ion source.

To quantitatively evaluate the improvement in diagnostic performance resulting from data dimensionality reduction and algorithm optimization, a systematic multi-model horizontal comparison is conducted under the same experimental parameters as previously described. The SSA-SVM and ISSA-SVM models are introduced to identify the ion source statuses. SSA and ISSA are employed to optimize the SVM parameters (C,g), constructing the SSA-SVM and ISSA-SVM models, respectively. This verifies the necessity of parameter optimization and the performance of the ISSA in discovering global optima and enhancing classification accuracy. Finally, the diagnostic results of these models are compared with those of the KPCA-ISSA-SVM model. The following sections detail the classification distributions and confusion matrices for the alternative models on the same test set, providing an in-depth analysis of overall accuracy and category-specific recognition rates. All results presented below represent the optimal performance from 30 independent runs.

**Figure 13:**
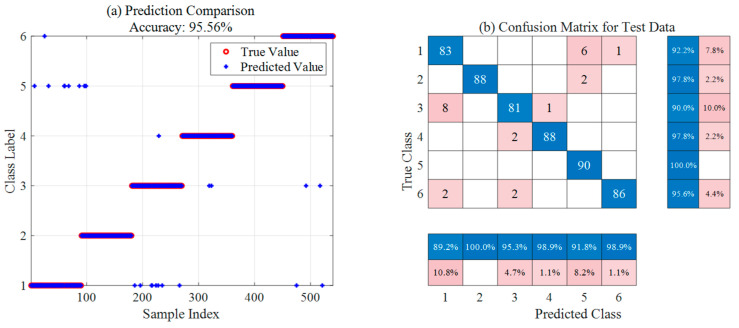
Optimal ion source diagnosis results based on SSA-SVM.

**Figure 14:**
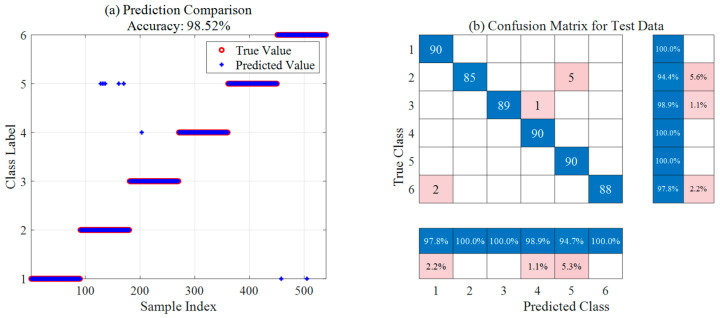
Optimal ion source diagnosis results based on ISSA-SVM.

Figure 13 and Figure 14 present the optimal results of the SSA-SVM and ISSA-SVM models on the ion source fault diagnosis test set, respectively, including prediction comparison plots and confusion matrices. The prediction comparison indicates that ISSA-SVM (98.52% accuracy) significantly outperforms SSA-SVM (95.56% accuracy) in overall classification precision, showing smaller deviations between predicted and true values. Based on the confusion matrix analysis, ISSA-SVM generally achieves higher accuracy across all fault categories. Notably, it exhibits substantial improvements in identifying samples from categories 1, 3, and 4, with a marked reduction in misclassified cases. These results demonstrate that the ISSA more effectively optimizes SVM parameters. Consequently, it enhances the classification performance and robustness of the model for ion source diagnosis, showcasing superior capability in discovering global optima.

Table 8 summarizes and compares the optimal diagnostic results of the three models across 30 independent runs. The experimental data indicate that the baseline SSA-SVM model achieves an overall accuracy of 95.56%, exhibiting certain limitations in identifying categories F1 and F3, which are susceptible to noise interference. With the introduction of the ISSA, the overall accuracy of ISSA-SVM significantly increases to 98.52%. Notably, the recognition rate for category F1 improves from 92.2% to 100.0%, demonstrating the superior performance of ISSA in discovering global optima and optimizing SVM parameters. Building upon this, the KPCA-ISSA-SVM model further eliminates redundant information from the raw data through feature dimensionality reduction, ultimately achieving an optimal classification accuracy of 99.63% and nearly perfect recognition across categories F1 to F5. In conclusion, the integration of KPCA and ISSA parameter optimization substantially enhances the classification performance and robustness, showcasing high reliability and engineering application value for diagnosing complex ion source operating statuses.

To impartially verify the fault diagnosis capability of the proposed model, this study follows several evaluation criteria. First, uniform experimental benchmarks are established: all comparative models are trained and tested using identical hardware configurations and raw datasets to eliminate biases caused by environmental factors. Second, statistical significance is emphasized. Due to the inherent randomness of swarm intelligence algorithms, each model undergoes 30 independent runs. The convergence stability and robustness of the algorithms are then assessed by comparing the average, optimal, and lowest accuracies. Finally, multi-dimensional evaluation metrics are introduced. Beyond classification precision, the computational overhead is also incorporated to comprehensively evaluate the engineering application potential of the models in real-time monitoring systems.

Table 9 presents the comprehensive performance evaluation results of different models in the ion source status recognition task, covering four metrics: average accuracy, optimal accuracy, lowest accuracy, and average execution time. The data indicate that the KPCA-ISSA-SVM model achieves the best performance among all compared methods. Specifically, its average accuracy reaches 97.6%, and its optimal accuracy is as high as 99.63%, which is significantly higher than those of other models such as Random Forest (RF), BP Neural Network, PSO-XGBoost, and PSO-Naive Bayes. Although the average execution time of KPCA-ISSA-SVM is 6 s, slightly higher than the 3.56 s of Random Forest, it maintains a clear advantage in recognition precision and stability. Furthermore, its lowest accuracy reaches 90.56%, demonstrating superior stability compared to the other models.

Table 10 compares the comprehensive performance of five classification models in the ion source status diagnosis task, with evaluation metrics including average accuracy, precision, recall, F1 score, and the Kappa coefficient. The data indicate that the KPCA-ISSA-SVM model achieves the best performance across all evaluation metrics. Specifically, its average accuracy reaches 97.6%, while its precision, recall, and F1 score are 0.978, 0.976, and 0.976, respectively. Notably, its Kappa coefficient reaches 0.971, demonstrating an extremely high level of consistency between the classification results and the actual labels. In contrast, Random Forest (RF) and the BP Neural Network show lower performance, with average accuracies of 88.53% and 84.37%, and Kappa coefficients of 0.862 and 0.812, respectively. Although the average accuracy of the PSO-XGBoost model reaches 94.03%, a certain gap still exists compared to the KPCA-ISSA-SVM. The PSO-Naive Bayes model exhibits the weakest overall performance, with an average accuracy of only 79.61%. Overall, the proposed model demonstrates significant advantages across multiple evaluation dimensions.

## 5. Conclusions

This study focuses on the ion source of CIAE’s 14 MeV cyclotron. Addressing the maintenance requirements under complex operating conditions, a KPCA-ISSA-SVM intelligent fault diagnosis model is systematically constructed and validated. This model effectively overcomes the challenges of difficult feature extraction and low classification accuracy. The primary research findings and core conclusions are summarized as follows:

(1) Dimensionality reduction effectively enhances data quality. In comparative validations with other classic dimensionality reduction algorithms, KPCA demonstrates excellent stability. It effectively addresses the curse of dimensionality and overfitting issues that traditional SVM models often encounter when handling high-dimensional data. Specifically, the model incorporating KPCA achieves an optimal accuracy of 99.63% across 30 independent tests, compared to 98.52% for the model without KPCA. This comparison proves that KPCA dimensionality reduction effectively raises the performance ceiling of the model.

(2) Multi-strategy optimization significantly enhances model performance. Considering the specific characteristics of accelerator fault data, the proposed ISSA-SVM model effectively improves diagnostic accuracy. Across multiple independent experiments, the optimal accuracy of ISSA-SVM is higher than that of the baseline SSA-SVM model. Notably, the recognition precision for minority class faults shows a significant improvement compared to the original SSA, which validates the effectiveness of the proposed optimization strategies.

(3) The integrated KPCA-ISSA-SVM model demonstrates high diagnostic precision and engineering value. In comparison with other commonly used fault models, the proposed KPCA-ISSA-SVM integrated diagnostic model exhibits the most outstanding performance. Across 30 independent test runs, its average accuracy reaches 97.6%. Furthermore, its worst-case result remains above 90%, demonstrating superior stability compared to other algorithms.

In summary, the research results demonstrate the high accuracy and efficiency of the KPCA-ISSA-SVM model in ion source fault diagnosis. This model provides robust technical support for the stable operation and intelligent maintenance of accelerators. Following the trend of series development in cyclotrons, future research focuses on the generalized application of this model in more complex scenarios, including multi-concurrent faults and cross-machine implementation. Continuous efforts also aim to optimize the computational overhead of the algorithm while enhancing the online monitoring and adaptive migration capabilities of the system. These advancements better meet the universal engineering requirements for real-time fault identification across various accelerator specifications in industrial environments.

## Figures and Tables

**Table 1:** List of input variables for the ion source fault diagnosis model.

Variables	Unit	Range (Min–Max)
Negative High-Voltage Power Supply Current	Kilo Volt (kV)	0–40
Negative High-Voltage Power Supply Voltage	Milli Ampere (mA)	0–75
Filament Power Supply Voltage	Volt (V)	0–15
Filament Power Supply Current	Ampere (A)	0–160
Arc Power Supply Voltage	Volt (V)	0–120
Arc Power Supply Current	Ampere (A)	0–30
Extraction Power Supply Voltage	Kilo Volt (kV)	0–4
Extraction Power Supply Current	Milli Ampere (mA)	0–100
Plasma Power Supply Voltage	Volt (V)	0–20
Plasma Power Supply Current	Ampere (A)	0–30
Hydrogen Gas Flow Rate	SCCM	0–20
Low Vacuum Gauge	mbar	$10^{3}-10^{-3}$
High Vacuum Gauge	mbar	$5\times 10^{-3}-5\times 10^{-8}$
Beam Current	mA	0–7

**Table 2:** Diagnostic performance comparison of SVM models with different parameters.

Parameters (*C*, *g*)	Overall Accuracy	Normal Recognition Rate (Class 1)	Fault Recognition Rate (Class 2)	Missed Faults
C=0.1, g=0.01	65.56%	78.9%	52.2%	62
C=1, g=0.1	71.67%	100.0%	43.3%	51
C=100, g=10	93.33%	100.0%	86.7%	12

**Table 3:** Performance comparison of SSA and ISSA under different population sizes.

Population Size	Algorithm	Avg. Time (s)	Mean/10^−3^	Optimal/10^−3^	Worst-Case/10^−3^
10	SSA-SVM	1.41	17.61	11.90	28.57
	ISSA-SVM	1.34	8.57	7.14	11.90
30	SSA-SVM	4.40	15.71	11.90	19.05
	ISSA-SVM	3.77	8.10	4.77	9.52
50	SSA-SVM	7.02	16.19	14.29	19.04
	ISSA-SVM	6.19	8.10	7.14	9.52
100	SSA-SVM	14.07	13.33	9.52	19.04
	ISSA-SVM	12.20	8.10	4.77	9.52

**Table 4:** Performance comparison of SSA and ISSA under different numbers of iterations.

Iterations	Algorithm	Avg. Time (s)	Mean/10^−3^	Optimal/10^−3^	Worst-Case/10^−3^
30	SSA-SVM	1.41	17.61	11.90	28.57
	ISSA-SVM	1.34	8.57	7.14	11.90
50	SSA-SVM	2.23	18.57	14.28	23.81
	ISSA-SVM	2.20	7.62	4.76	9.52
100	SSA-SVM	4.63	19.05	14.29	21.43
	ISSA-SVM	4.53	8.09	4.76	9.52

**Table 5:** Comparison of ion source status diagnosis results between SSA-SVM and ISSA-SVM.

Model	Overall Accuracy	Normal Recognition Rate (Class 1)	Fault Recognition Rate (Class 2)	Missed Faults
SSA-SVM	96.11%	100.0%	92.2%	7
ISSA-SVM	99.44%	100.0%	98.9%	1

**Table 6:** Coding of ion source fault types.

Status Code	Status Category
F1	Normal operation
F2	Plasma sparking
F3	Extraction sparking
F4	Simultaneous plasma and extraction sparking
F5	Filament collapse sparking
F6	Abnormal ion source status

**Table 7:** Comprehensive evaluation of ion source status recognition performance based on different dimensionality reduction algorithms.

Algorithm	Average Accuracy (%)	Optimal Accuracy (%)	Lowest Accuracy (%)	Average Time (s)
KPCA	97.60	99.63	90.56	6.56
LDA	88.78	99.26	48.89	6.11
PCA	95.11	99.26	45	6.22
t-SNE	92.24	98.33	51.85	7.15
Isomap	65.05	72.96	47.96	26.75

**Table 8:** Comparison of optimal ion source diagnosis results among different models across 30 independent runs.

Sample Category	Quantity	SSA-SVM	ISSA-SVM	KPCA-ISSA-SVM
		Misclassified/ Accuracy (%)	Misclassified/ Accuracy (%)	Misclassified/ Accuracy (%)
F1	90	7/92.2%	0/100.0%	0/100.0%
F2	90	2/97.8%	5/94.4%	0/100.0%
F3	90	9/90.0%	1/98.9%	0/100.0%
F4	90	2/97.8%	0/100.0%	1/98.9%
F5	90	0/100.0%	0/100.0%	0/100.0%
F6	90	4/95.6%	2/97.8%1/	1/98.9%
F1–F6	540	24/95.56%	8/98.52%	2/99.63%

**Table 9:** Comprehensive evaluation of ion source status recognition performance based on different models.

Algorithm	Average Accuracy (%)	Optimal Accuracy (%)	Lowest Accuracy (%)	Average Time (s)
KPCA-ISSA-SVM	97.60	99.63	90.56	6
Random Forest (RF)	88.53	93.52	82.78	3.56
BP Neural Network	84.37	90.37	73.89	26.40
PSO-XGBoost	94.03	95.19	89.26	18.34
PSO-Naive Bayes	79.61	82.22	63.89	6.46

**Table 10:** Diagnostic performance comparison of different classification models across multiple evaluation metrics.

Algorithm	Average Accuracy (%)	Precision	Recall	F1 Score	Kappa
KPCA-ISSA-SVM	97.6	0.978	0.976	0.976	0.971
Random Forest (RF)	88.53	0.891	0.887	0.884	0.862
BP Neural Network	84.37	0.852	0.842	0.841	0.812
PSO-XGBoost	94.03	0.942	0.942	0.939	0.929
PSO-Naive Bayes	79.61	0.805	0.793	0.793	0.755

## Data Availability

The data presented in this work are available from the corresponding author upon reasonable request.
